# Clinical and Cognitive Improvement Following Treatment with a Hemp-Derived, Full-Spectrum, High-Cannabidiol Product in Patients with Anxiety: An Open-Label Pilot Study

**DOI:** 10.3390/biomedicines13081874

**Published:** 2025-08-01

**Authors:** Rosemary T. Smith, Mary Kathryn Dahlgren, Kelly A. Sagar, Deniz Kosereisoglu, Staci A. Gruber

**Affiliations:** 1 Cognitive and Clinical Neuroimaging Core (CCNC), McLean Hospital, Belmont, MA 02478, USA; rsmith@mclean.harvard.edu (R.T.S.); dahlgren@mclean.harvard.edu (M.K.D.); ksagar@mclean.harvard.edu (K.A.S.); dkosereisoglu@mclean.harvard.edu (D.K.); 2 Marijuana Investigations for Neuroscientific Discovery (MIND) Program, McLean Hospital, Belmont, MA 02478, USA; 3 Department of Psychiatry, Harvard Medical School, Boston, MA 02115, USA

**Keywords:** anxiety, cannabidiol, hemp, cannabis

## Abstract

**Background/Objectives**: Cannabidiol (CBD) is a non-intoxicating cannabinoid touted for a variety of medical benefits, including alleviation of anxiety. While legalization of hemp-derived products in the United States (containing ≤0.3% delta-9-tetrahydrocannabinol [d9-THC] by weight) has led to a rapid increase in the commercialization of hemp-derived CBD products, most therapeutic claims have not been substantiated using clinical trials. This trial aimed to assess the impact of 6 weeks of treatment with a proprietary hemp-derived, full-spectrum, high-CBD sublingual solution similar to those available in the marketplace in patients with anxiety. **Methods**: An open-label pilot clinical trial (NCT04286594) was conducted in 12 patients with at least moderate levels of anxiety. Patients self-administered a hemp-derived, high-CBD sublingual solution twice daily during the 6-week trial (target daily dose: 30 mg/day CBD). Clinical change over time relative to baseline was assessed for anxiety, mood, sleep, and quality of life, as well as changes in cognitive performance on measures of executive function and memory. Safety and tolerability of the study product were also evaluated. **Results**: Patients reported significant reductions in anxiety symptoms over time. Concurrent improvements in mood, sleep, and relevant quality of life domains were also observed, along with stable or improved performance on all neurocognitive measures. Few side effects were reported, and no serious adverse events occurred. **Conclusions**: These pilot findings provide initial support for the efficacy and tolerability of the hemp-derived, high-CBD product in patients with moderate-to-severe levels of anxiety. Double-blind, placebo-controlled studies are indicated to obtain robust data regarding efficacy and tolerability of these types of products for anxiety.

## 1. Introduction

Anxiety is consistently noted as one of the most common symptoms for which patients report using medical cannabis (MC) and cannabinoid-based therapies [1,2]. As some of the most common psychiatric conditions, affecting over a third of adults in the United States (U.S.) [3], anxiety disorders are often difficult to treat; many patients experience incomplete symptom relief, delayed treatment response, and bothersome side effects [4,5,6,7]. There is a clear need for novel treatment options that are likely to result in full remission of anxiety symptoms with improved tolerability over current treatment options.

Cannabis has been used medicinally for a range of disorders for thousands of years [8], and more recently researchers have sought to determine conditions for which cannabis and its constituents may provide clinical benefit [9]. In the U.S., the majority of states have fully legalized MC programs, while almost all other states allow limited use of certain CBD products for specific indications. Additionally, under the U.S. Agricultural Improvement Act of 2018 (known as the Farm Bill), products derived from industrial hemp (defined as ≤0.3% delta-9-tetrahydrocannabinol (d9-THC) by weight) are now excluded from the Controlled Substance Act, and thereby legal in the U.S. [10]. Although cannabis is often identified by d9-THC (its main intoxicating compound), many consumers are interested in products with varied constituent profiles, including non-intoxicating cannabinoids with therapeutic potential [11].

Cannabidiol (CBD), the most widely known non-intoxicating cannabinoid, appears to have potential therapeutic benefits for a range of conditions, including anxiety [2]. A number of preclinical studies have provided evidence of anxiolytic effects following both acute and chronic administration of CBD [12]. While few human studies have been conducted to investigate the impact of CBD on anxiety, compelling evidence indicates that CBD may exert a potent anxiolytic effect, mirroring animal studies. A recent meta-analysis of eight clinical investigations indicated CBD may significantly improve anxiety, but more clinical trials are necessary [13]. Several acute administration studies using the simulated public speaking test (SPST), a model of experimentally induced anxiety, have demonstrated reductions in anxiety in both healthy volunteers [14] and patients with social anxiety disorder (SAD) [15,16] following administration of 300–600 mg of CBD. More recently, a double-blind clinical trial in adolescents with SAD reported significant reductions in anxiety following 4 weeks of treatment with 300 mg/day of CBD [17], and an open-label 12-week clinical trial in adolescents and young adults with treatment-resistant anxiety reported improvements in anxiety and depressive symptoms following treatment with up to 800 mg/day of CBD [18].

Importantly, these studies utilized a CBD isolate, without other cannabinoids or compounds; several studies have suggested that isolates are less effective than products containing a range of naturally occurring compounds (multiple cannabinoids, terpenes, flavonoids) [19,20,21]. Full-spectrum products contain a wide range of cannabinoids, including d9-THC, while broad-spectrum products contain multiple cannabinoids except for d9-THC. Full- and broad-spectrum cannabinoid products are prevalent in the marketplace, but few studies have assessed the impact of these products. In addition, previous studies have largely focused on patients with SAD, not those with generalized anxiety. Recently, our group conducted an open-label [22] to double-blind clinical trial of a cannabis-derived, full-spectrum, low-THC, high-CBD product in patients with moderate-to-severe anxiety and reported significant improvement on measures of anxiety, mood, quality of life, sleep, and executive function after 4 weeks of treatment with 30 mg CBD/day (10 mg/mL) using a three times per day (TID) schedule. As that trial used a much lower dose of CBD than previous investigations using CBD isolate (e.g., 30 mg/day vs. 300–800 mg/day), findings provided additional evidence that lower doses of full-spectrum, high-CBD products may be efficacious for treating anxiety and may result in concurrent improvements in other domains, including executive function. While this trial provided preliminary evidence for the efficacy of lower doses of full-spectrum, cannabis-derived CBD products for moderate-to-severe anxiety, the impact of a hemp-derived product was yet unknown.

The aim of the current open-label clinical trial is to assess the impact of a hemp-derived, full-spectrum, high-CBD sublingual study product, formulated to be similar in dose and cannabinoid profile to many products available in the marketplace, in patients with at least moderate levels of anxiety over 6 weeks of treatment (NCT04286594). Patients self-administered 0.5 mL of sublingual solution containing approximately 30 mg/mL CBD twice daily (BID), for a target daily dose of 30 mg CBD. Given previous research indicating potential anxiolytic effects of high-CBD products, we hypothesized that patients would demonstrate reductions in anxiety along with clinical improvement of related symptoms (e.g., mood) and quality of life following 6 weeks of treatment with the study product. Further given our previous work indicating that high-CBD regimens are not associated with cognitive decrements, we expected that patients would demonstrate stable or improved performance on measures of executive function and memory.

## 2. Materials and Methods

### 2.1. Ethical Approval

This study was approved by the Mass General Brigham Institutional Review Board (protocol 2019P003885) and carried out in accordance with the Declaration of Helsinki. Patients provided written informed consent to voluntarily participate in study procedures.

### 2.2. Study Design and Participants

Study enrollment was completed at McLean Hospital in Belmont, Massachusetts, U.S. between February 2021–August 2023. All patients completed a baseline visit with follow-up visits occurring weekly throughout the 6-week trial (7 visits total) and were compensated $350 for completing the study.

This pilot, proof-of-concept, open-label trial was designed to enroll 12 patients who completed the trial. The sample size for this pilot study was determined based on results from the open-label phase of our ongoing clinical trial assessing the impact of a full-spectrum, high-CBD sublingual solution (10 mg CBD TID for total daily dose of 30 mg) on anxiety symptoms [22]. The open-label pilot phase of that trial had a sample size of 14 patients and demonstrated very large effect sizes for the primary outcome variables (Beck Anxiety Inventory [BAI] η_p_^2^ = 0.65 and Overall Anxiety Severity and Impairment Scale [OASIS] η_p_^2^ = 0.75). Conservative power analyses using the threshold for large effects (η_p_^2^ = 0.14) at 95% power and α = 0.05 indicated a total sample size of at least 11 patients was required. Given the extremely large effect sizes from the previous trial, and given that the present study was designed to closely mirror the previous trial but using hemp instead of a cannabis-derived study product, the sample size of the current trial was considered sufficiently powered.

Patients reporting at least moderate levels of anxiety, defined as ≥16 on the BAI [23] or ≥8 on the OASIS [24] at baseline, were recruited from the greater Boston area through online advertisements and flyers. Patients were required to have a stable pharmacotherapeutic regimen for at least three months prior to enrollment and current use of cannabis or cannabinoid products was not allowed (i.e., at baseline, all patients were required to test negative for d9-THC metabolites and use cannabis no more than 1×/month). Use of cannabinoid products other than the study product was not allowed during the trial. In addition, individuals were required to have an IQ > 75, as assessed by the 2-factor Wechsler Abbreviated Scale of Intelligence—II (WASI-II) [25], to be included in the study.

### 2.3. Study Product

The high-CBD study product contained full-spectrum extract of a proprietary hemp genetic (CW1AS1) from Charlotte’s Web (CW) that was custom formulated in medium chain triglyceride (MCT) oil and dispensed into 30 mL glass bottles for the study. The final formulation contained 31.52 mg/mL CBD and 0.77 mg/mL THC, along with small amounts of several other cannabinoids (see Table 1), confirmed via liquid chromatography (LC) by ProVerde Laboratories (Milford, MA, USA). Twice a day throughout the 6-week trial, patients were instructed to deposit 0.5 mL of solution under-the-tongue and hold for a minimum of 60 s before swallowing. The target daily dose was 30 mg CBD per day. Compliance was assessed using drug diaries, and study product use was quantified by cross-referencing drug diaries with bottle weights obtained at each visit. Further, patients provided urine samples every other week which were used to obtain an in-house “positive” or “negative” reading for d9-THC (CLIAwaived, Inc. [Carlsbad, CA, USA] 12-Panel IDTC Cups, including a panel of common drugs with high abuse potential); all samples were also sent to Quest Diagnostics for gas chromatography-mass spectrometry quantification (GC-MS) of THC-COOH, a primary metabolite of d9-THC, which were creatinine-corrected similar to previous work [26].

### 2.4. Clinical Scales

All patients completed the Structured Clinical Interview for DSM-5 Research Version (SCID-5-RV) [27] at their baseline visit. At each weekly visit, patients completed a clinical check-in with study staff and several rating scales assessing various aspects of anxiety. The BAI, a brief self-report measure of anxiety symptoms, was used to assess study eligibility and served as the primary outcome variable [23]. The OASIS, also used to determine study eligibility, was used to assess changes in self-reported anxiety-related symptoms and impairment due to anxiety [28]. The State-Trait Anxiety Inventory (STAI) was completed to differentiate between potential changes in patients’ current levels of anxiety (STAI-State) and general levels of anxiety (STAI-Trait) [29]. Finally, the Hamilton Anxiety Rating Scale [HAM-A], a clinician-rated measure less likely to be biased by self-report, was used to assess severity of anxiety symptoms at each visit [30].

Patients also completed weekly self-report ratings of mood including the Beck Depression Inventory (BDI [31]), Positive and Negative Affect Scale (PANAS [32]), and the Profile of Mood States (POMS [33]) which consists of several subscales that generate a composite score reflecting “total mood disturbance” (TMD). At the baseline and 6-week visits, the Pittsburgh Sleep Quality Index (PSQI [34]) was used to assess sleep disturbance, and quality of life was measured using the Medical Outcomes Study Questionnaire Short Form 36 Item (SF-36 [35]), which provides an 8-scale profile detailing overall well-being and health.

In addition, a treatment expectancy questionnaire was administered at baseline to assess the extent to which patients believed the study product would impact anxiety; patients rated how they thought the study product would impact their anxiety on a scale from 1 (“It will make my anxiety significantly worse”) to 5 (“It will make my anxiety significantly better”). A modified version of this scale was also completed at the final study visit to assess actual perceived effects of the study product (i.e., what impact do you think the study product has had on your anxiety?). In addition, at the 6-week visit, the Patient’s Global Impression of Change (PGIC [36]) was also administered to assess patients’ perception of change from baseline; the scale ranged from 1 (“no change or condition has gotten worse”) to 7 (“a great deal better and a considerable improvement that has made all the difference”).

An Abuse Liability Rating scale (ALR) was administered at week 6, querying the strength of the product, good/bad effects, how much the product was liked, willingness to take again, and whether the patient felt any intoxication; ALR responses were on a scale of 0 (no effect/not at all) to 4 (extremely). At each follow-up visit, patients also completed a Side Effects Questionnaire (SEQ) adapted by our lab from commonly administered adverse event rating scales in order to include cannabis-specific items (e.g., feeling “altered”, etc.) while also querying a multitude of potential side effects and tolerability (total number of items = 42). Endorsed side effects were classified as positive, neutral, or negative in nature in order to separate unwanted side effects from more positive treatment effects, with the extent of the effect rated using a 3-point scale (minimum, moderate, or maximum). All endorsed side effects that were rated as moderately or maximally negative were recorded as adverse events.

### 2.5. Cognitive Assessments

At baseline and 6-week visits, patients completed a comprehensive neuropsychological battery, primarily designed to interrogate executive function and memory, domains shown to be significantly impacted by cannabis use [37,38]. Executive function was assessed using the Stroop Color-Word Test [39], Trail Making Test (TMT [40]), the Multi Source Interference Test (MSIT [41]), the Letter-Number Sequencing (LNS) subtest of the Wechsler Adult Intelligence Scale-Revised (WAIS-R [42]), and the Controlled Oral Word Association Test (COWAT [43]). Visual memory was assessed using the Benton Visual Retention Test (BVRT [44]), while verbal learning and memory were assessed using the Rey Auditory Verbal Learning Test (RAVLT [45]). Alternate versions of each task were used at the 6-week visit to limit the impact of practice effects.

### 2.6. Statistics

Data were collected and managed using REDCap [46,47] and analyzed using SPSS v.28 (α = 0.05, two-tailed tests). Descriptive statistics were conducted for data collected at a single time point (e.g., demographics, ALR, PGIC, etc.) as well as for the urinalysis data. Since one patient did not complete the 5-week visit due to travel, inferential analyses without listwise deletion were selected; all other patients completed each weekly visit. Linear mixed model (LMM) analyses with first-order autoregressive AR(1) covariance structures (reference group = baseline) were conducted to assess mean change in continuous outcome variables from baseline. The BAI, OASIS, HAM-A, STAI, BDI, POMS, and PANAS scores were assessed weekly over the course of the 6-week trial while the PSQI, SF-36, and all cognitive assessments were assessed only at baseline and week 6. The estimates of the fixed effects from the LMMs provided information regarding significant change over time for each time point relative to baseline. In order to ensure the residuals from the LMM analyses were normally distributed, several checks of normality were completed. Lilliefors-corrected Kolmogorov–Smirnov and Shapiro–Wilk tests were conducted to assess the distribution of the residuals from the LMM analyses; however, given limitations of these tests [48], Q-Q plots of the residuals as well as assessments of skew and kurtosis were also evaluated. These assessments indicated no concern for violating the assumption of normality required for LMM assessments.

Treatment expectancy data were collected with the intent to use expectancy as a covariate in the LMM analyses; however, homogeneity of ratings indicated treatment expectancy was not an appropriate covariate (i.e., inclusion in the statistical models was similar to including a constant). Pearson’s correlations assessed the linear relationship between positive and negative treatment expectancies and the percent improvement of any clinical variable that changed significantly following treatment. These correlations and the scatterplots of their distributions confirmed that the homogeneity of these ratings was problematic (i.e., patient treatment expectancies were all highly ranked with little variance); therefore, covariate and correlation analyses of treatment expectancy were inappropriate due to restricted range.

In addition, percent change relative to baseline was calculated for the BAI for all follow-up timepoints in order to identify frequencies of treatment responders, defined as those achieving and maintaining clinical improvement of ≥15% reduction in BAI scores. This threshold was identified as being optimal to identify treatment responders [49,50], and is consistent with thresholds used in our previous work [22]. Interim analyses were conducted after the first 5 patients completed the open-label trial in order to assess clinical response (based on BAI scores) to confirm adequate dosing; interim analyses revealed that this threshold was met and no changes in dosing were required.

## 3. Results

### 3.1. Participant Flow and Demographics

Ninety-three patients were assessed for eligibility. Fifteen patients were consented and enrolled, and three patients were disqualified or discontinued during the first visit (one did not meet minimum anxiety criteria, one was not fluent in English, and one exited the remote baseline visit and was unable to be recontacted). Twelve patients (9 females, 3 males) aged 22–64 years (average: 38.00 ± 12.14) completed the full 6-week trial. All enrolled patients met DSM-5 criteria for current generalized anxiety disorder (GAD) based on the SCID-5-RV. Table 1 presents the characteristics of the study sample.

Information was also collected regarding cannabinoid use histories, which revealed that 11 of 12 patients had a past history of trying or using cannabis or cannabinoid products. Specifically, 7 tried cannabis fewer than 30x in their lifetime (average of 14 uses). The other 4 patients reported a past history of regular recreational cannabis use (up to 2×/week); however, average duration of abstinence since last use was 6.33 years.

### 3.2. Study Product Use

Drug diaries documenting product use, which were corroborated by weighing the study product bottles at each visit, indicated patients used an average daily dose of 31.29 ± 6.32 mg CBD; the average daily dose of CBD was similar to the target dose of 30 mg/day. Total days of treatment with the study product ranged from 39 to 56 days (target: 42 days). Correlation analyses between number of treatment days and percent change in clinical rating scales were non-significant, except for a negative correlation between number of treatment days and the General Health subscore of the SF-36 (*r* = −0.686, *p* = 0.014).

Despite the small amount of d9-THC contained in the study product (0.77 mg/mL), in-house urine assays indicated that 25% of patients at weeks 2 and 4 and 33.3% of patients at week 6 tested positive for d9-THC (Table 2). Further, more sensitive gas chromatography-mass spectrometry (GC-MS) quantification of creatinine-corrected urinary THC-COOH was positive in 50% of patients at each visit, although it remained relatively low (average 11–12 ng/mg).

### 3.3. Clinical State and Treatment Response

**Figure 1:**
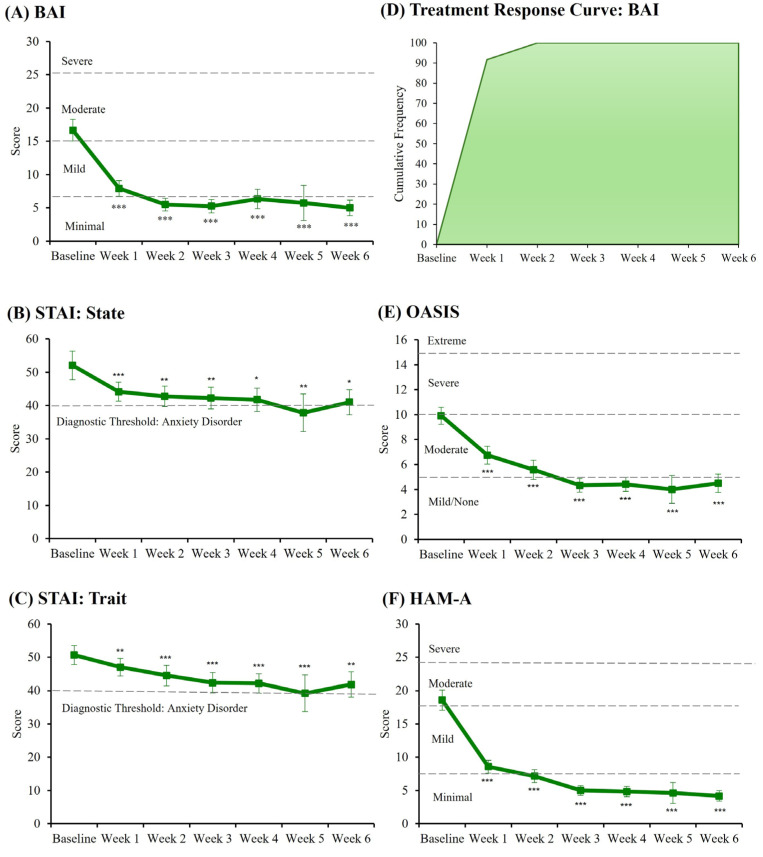
Line graphs of anxiety ratings and cumulative frequency of treatment responders over 6 weeks. Significant reductions in anxiety following 6 weeks of treatment relative to baseline were noted for the (**A**) Beck Anxiety Inventory (BAI), (**B**) State-Trait Anxiety Inventory: State (STAI: State), (**C**) State-Trait Anxiety Inventory: Trait (STAI: Trait), (**E**) Overall Anxiety Severity and Impairment Scale (OASIS), and (**F**) Hamilton-Anxiety Rating Scale (HAM-A). Clinical thresholds for each scale are indicated by dashed lines. In addition, a cumulative frequency curve of treatment responders on the BAI (**D**) indicates those who achieved and maintained clinical improvement of at least 15% reduction in BAI ratings relative to baseline. Significant contrasts are noted as: * *p* ≤ 0.050; ** *p* ≤ 0.010; *** *p* ≤ 0.001.

Following 6 weeks of treatment, significant decreases were noted relative to baseline on all scales assessing anxiety (Table S1), including self-report measures BAI (*p* < 0.001), OASIS (*p* < 0.001), STAI-State (*p* = 0.022), STAI-Trait (*p* = 0.005), and the clinician-rated HAM-A (*p* < 0.001) (Figure 1). Notably, all anxiety ratings were significantly reduced following 1 week of treatment; 91.67% of patients achieved ≥15% reduction on the BAI indicating treatment response following 1 week, and 100% of patients achieved and maintained treatment response following 2 weeks of treatment (Figure 1).

Patients also exhibited significant improvements on assessments of mood, sleep, and quality of life after 6 weeks of treatment. Specifically, patients reported reduced symptoms of depression on the BDI (*p* < 0.001), increased positive affect (*p* = 0.003) and reduced negative affect (*p* < 0.001) on the PANAS, and improved mood on the POMS TMD (*p* = 0.017). Patients also reported improved sleep quality on the PSQI (*p* < 0.035) and higher quality of life ratings on several subscales of the SF-36, including emotional problems (*p* = 0.011), energy/fatigue (*p* < 0.001), emotional well-being (*p* = 0.001), and social functioning (*p* = 0.020). The complete statistical results of the clinical state analyses are presented in Supplementary Materials (Table S1).

### 3.4. Expectancy and Perceived Effects

On the treatment expectancy scale, all patients indicated positive expectancies at baseline, either selecting “it will make my anxiety a little better” (*n* = 9, 75.0%) or “it will make my anxiety significantly better” (*n* = 3, 25.0%).

After 6 weeks of treatment, all patients reported positive perceived effects of the study product on anxiety, either selecting “it has made my anxiety a little better” (*n* = 7, 58.3%) or “it has made my anxiety significantly better” (*n* = 5, 41.7%). On the PGIC, patients also reported a median score of 5.00 (range: 4–7), indicating they described “the change (if any) in activity limitations, symptoms, emotions, and overall quality of life” as “moderately better, and a slight but noticeable change”.

### 3.5. Side Effects and Tolerability

The study drug was well-tolerated, with no serious adverse events occurring during the trial. Overall, responses on the ALR (Table 1) suggest that patients liked the product and were willing to take it again; patients also reported experiencing moderately good effects with virtually no bad effects. Three adverse events (AEs) were recorded throughout the trial, derived from SEQ items that were rated moderately or maximally negative; these AEs included moderately decreased libido (*n* = 1, 8.33%), moderate nausea (*n* = 1, 8.33%), and moderate weight gain (*n* = 1, 8.33%). No maximally negative side effects were reported.

### 3.6. Cognitive Tasks

Following 6 weeks of treatment, patients demonstrated significantly improved performance on measures of executive function relative to baseline, evidenced by faster response time on the interference conditions of the MSIT (*p <* 0.001) as well as higher accuracy (*p =* 0.002) compared to baseline; improvement was also noted on response time and accuracy in the control condition (*p =* 0.015 and *p =* 0.026, respectively). Additionally, patients demonstrated faster response time on Trails A of the TMT (*p =* 0.034) and stable response time on Trails B of the TMT after 6 weeks of treatment compared to baseline (Table 3). Performance on other tasks of executive function (Stroop, LNS, COWAT) remained stable, as did visual memory (BVRT) and verbal learning and memory (RAVLT).

## 4. Discussion

Findings from this clinical trial provide preliminary evidence that use of this proprietary hemp-derived, full-spectrum, high-CBD sublingual product may result in clinical improvement with few side effects in patients with moderate-to-severe anxiety, extending previous work suggesting CBD may be efficacious for anxiety [2,12,13,17,22,51]. In the present study, dramatic reductions in anxiety occurred following just one week of treatment with the study product, which is particularly notable as many first-line treatments for anxiety (e.g., selective serotonin reuptake inhibitors [SSRIs]) can take several weeks to demonstrate efficacy [52]. Interestingly, reductions in anxiety and mood-related symptoms were noted on both self-report and clinician-driven assessments; clinician-administered scales are less prone to bias than self-report measures, strengthening findings from this trial. The present study also complements data from the open label phase of our previous clinical trial using a low d9-THC (0.29 mg/mL) cannabis-derived (vs. hemp-derived) sublingual high-CBD solution with a slightly different cannabinoid constituent profile and dosing schedule (BID in the present study vs. TID in the previous study, identical daily target dose of CBD). Patients in both studies reported similar improvements in anxiety, mood symptoms, sleep, and quality of life over 4 weeks of treatment, with large reductions in anxiety occurring after one week of treatment in both studies. Further, the same pattern of cognitive performance on measures of executive function and memory were observed (i.e., stable/improved performance on measures of executive function, stable performance on measures of memory) [22]. Interestingly, not all quality of life domains demonstrated change over time in the present study; specifically, domains for emotional problems, energy/fatigue, emotional well-being, and social functioning all significantly improved over the course of the trial, while physical functioning, physical health, pain, and general health domains remained stable. Importantly, however, in a clinical trial designed to address symptoms of anxiety, changes in domains reflecting physical or general health were not expected. Taken together, these studies demonstrate that full spectrum CBD-based products may provide symptom relief in patients with anxiety.

One strength of this study is the use of a product similar in composition to many hemp-derived cannabinoid products available throughout the U.S., adding ecological validity to the study. The study product created for this clinical trial was designed to be a combination of the original CW 50 mg CBD/mL and 17 mg CBD/mL sublingual oils, with daily dosing (~30 mg/day) intended to approximate the midpoint of these two doses. Within the commercial marketplace, hundreds of products containing CBD exist, both broad- and full-spectrum. This product was specifically formulated to be similar to some of the most common options across the marketplace to ensure it approximates those which are being used by patients in the ‘real world’. This is particularly important given increasing numbers of patients exploring these products on their own and also provides useful data to health care providers and clinicians in practice. While the current findings provide at least preliminary evidence that well-characterized, high CBD containing, hemp-derived full spectrum products are efficacious for symptoms of anxiety, additional research including larger scale, placebo-controlled, double blind clinical trials with custom formulated products must be completed in order to provide specific recommendations for clinical practice.

It is of note that despite the study product containing low levels of d9-THC, patients generally did not report feelings of intoxication. Interestingly, a single patient endorsed moderate intoxication on the ALR; however, their ratings for the corresponding item on the SEQ were generally classified as “neutral” in nature and therefore did not rise to the level of an AE. Notably, however, in-house assays indicated the presence of THC in a third of patients at week 6 (N = 4), and GC-MS analyses indicated quantifiable THC-COOH in 50% of patients (N = 6) at follow-up visits where urine was collected. These findings also align with our previous work demonstrating 50% of patients tested positive for THC-COOH following 4 weeks of treatment with a similar high-CBD study product [53], and underscore that even low levels of d9-THC in hemp-derived products can result in positive urine tests without patients experiencing intoxicating effects. Despite the fact that hemp-derived products are federally legal in the U.S., a positive urine screen could result in serious legal or job-related consequences.

In the present study, findings of stable or improved cognitive performance were largely consistent with data from previous observational studies of MC patients [54,55,56,57,58] and previous clinical trials and acute administration studies of CBD products [16,22,58,59]. Interestingly, these findings stand in contrast to a substantial body of previous research documenting decrements in cognitive function among recreational cannabis users [38], a discrepancy likely explained by several important factors. For example, the goal of use drives product selection, resulting in different cannabinoid profiles in products used (e.g., recreational products are typically high in THC and low in CBD). Age of onset of use is another critical differentiating variable, as cannabis use during developmentally vulnerable periods (e.g., adolescence) is associated with cognitive decrements [38,60]; importantly, this study included adults well beyond the period of neurodevelopmental vulnerability. In addition, previous work has indicated that in MC patients, cannabis use variables were not directly associated with cognitive improvement; instead, clinical improvement on measures of anxiety, mood, and sleep quality were significantly correlated with improved cognitive performance [56]. Taken together, it is likely that improved cognitive performance occurs secondary to clinical improvement, once clinical symptoms (including anxiety) are alleviated.

### Limitations

Limitations of the current study include small sample size; as a proof-of-concept study, only 12 patients were targeted for completion of this trial. Patients in the current study were demographically similar, with all patients reporting their race as White and non-Hispanic. The sample was also predominantly female (75%) with a relatively high level of education. While previous studies have indicated that women are more likely to have anxiety disorders than men [3], and Americans who are White have higher rates of anxiety compared to non-White individuals [61], additional studies with larger, more diverse samples are critical to determine generalizability of study findings. In addition, the sample was limited to individuals who are not currently using cannabinoid-based products, and who do not have extensive cannabis use histories, which may not be broadly generalizable.

It is important to note that the sublingual high-CBD solution formulated for this trial included small amounts of several other cannabinoids and a range of terpenes which may also have biobehavioral effects [62]. To date, it remains unclear whether CBD itself or a combination of CBD with other cannabinoids and terpenes may have led to the improvements in anxiety and mood demonstrated in this trial. Additional studies should explore this possibility.

Further, the open-label nature of this trial may have resulted in a significant placebo effect, as patients expect to feel better knowing that they are receiving an active study product. While expectancies were assessed in the current study, homogeneity of responses (e.g., all patients endorsed positive expectancy for MC and cannabinoid-based products to improve anxiety), made it impossible to covary for expectancy. In addition, patients reported positive perceived effects of the study product, and attributed improvements in their anxiety to the study product, suggesting self-reported clinical ratings may be subject to bias. As noted, future studies should include larger scale, randomized, double-blind designs in order to determine the impact of treatment expectancy and placebo effects.

Finally, number of treatment days ranged fairly significantly across patients, due to accommodating study visits around travel and other personal constraints. Importantly, however, correlations between percent change and number of treatment days were largely non-significant; the only clinical measure significantly associated with number of treatment days was the General Health subscore of the SF-36. Notable improvements in clinical state occurring after one week of the study product also minimize concerns regarding variability in dosing and treatment duration.

## 5. Conclusions

Results from this open-label clinical trial provide evidence that a hemp-derived, full-spectrum, high-CBD product similar to those currently available in the marketplace may be both safe and efficacious for the treatment of anxiety. Given the potential benefits observed in this trial, double-blind, placebo-controlled studies of hemp-derived high-CBD products are warranted to obtain robust data regarding the safety and efficacy of CBD-containing products for anxiety.

## Figures and Tables

**Table 1:** Demographics.

Demographics	Patients (*n* = 12)		
	*n* (%) or Mean ± SD	Minimum	Maximum
Sex:			
Female	9 (75.0%)	-	-
Male	3 (25.0%)	-	-
Age	38.00 ± 12.14	22	64
Estimated IQ (WASI)	109.75 ± 9.12	89	123
Body Mass Index (BMI)	26.40 ± 4.64	20.80	39.31
Race/Ethnicity:			
White, Non-Hispanic	12 (100.0%)	-	-
**High-Cannabidiol (CBD) Study Product Use**			
Treatment Days	44.92 ± 6.23	39	56
Product Use (mL/day)	0.97 ± 0.20	0.73	1.38
Exposure to Specific Cannabinoids ^a^ (mg/day):			
Cannabidiol (CBD)	31.29 ± 6.32	23.66	44.64
∆9-Tetrahydrocannabinol (d9-THC)	0.59 ± 0.12	0.45	0.85
Cannabichromene (CBC)	1.47 ± 0.30	1.11	2.09
Cannabigerol (CBG)	0.38 ± 0.08	0.29	0.54
Cannabidivarin (CBDV)	0.24 ± 0.05	0.18	0.35
Cannabidiolic acid (CBDA)	0.12 ± 0.02	0.09	0.17
**Abuse Liability Rating Scale (ALR) Following 6 Weeks of Treatment ^b^**			
Strength of Product	1.75 ± 0.75	1	3
Good Effects of Product	2.67 ± 1.07	1	4
Bad Effects of Product	0.25 ± 0.45	0	1
Liking Product	3.42 ± 0.67	2	4
Willingness to Take Again	3.83 ± 0.39	3	4
Feelings of Intoxication ^c^	0.17 ± 0.58	0	2

Abbreviations: WASI, Wechsler Abbreviated Scale of Intelligence. ^a^ The study product was also assessed for cannabinol (CBN), cannabigerolic acid (CBGa), tetrahydrocannabinolic acid (THCa), and tetrahydrocannabivarin (THCV), which were not present above the limit of detection; ^b^ ALR response options by item: 1. strength of product: 0—no effect, 1—a little, 2—moderately, 3—quite a bit, 4—extremely; 2. good effects of product: 0—not at all, 1—a little, 2—moderately, 3—quite a bit, 4—extremely; 3. bad effects of product: 0—not at all, 1—a little, 2—moderately, 3—quite a bit, 4—extremely; 4. liking product: 0—disliked very much, 1—disliked somewhat, 2—neutral, 3—liked somewhat, 4—liked very much; 5. willingness to take again: 0—very unwilling, 1—somewhat unwilling, 2—neutral, 3—somewhat willing, 4—very willing; 6. intoxication: 0—not at all, 1—a little, 2—moderately, 3—quite a bit, 4—extremely; ^c^ only one patient reported a non-zero response for this item.

**Table 2:** Urinalysis changes over 6 weeks of treatment with a high-CBD sublingual product (2-tailed).

	Baseline *n* = 12	2 Weeks *n* = 12	4 Weeks *n* = 12	6 Weeks *n* = 12
**Urine Cup Assay**	***n* (%)**	***n* (%)**	***n* (%)**	***n* (%)**
THC Positive ^a^	0 (0.0%)	3 (25.0%)	3 (25.0%)	4 (33.3%)
**Gas Chromatography-Mass Spectrometry Urinalyses (Quest Laboratories)**	***n* (%)**	***n* (%)**	***n* (%)**	***n* (%)**
	0 (0.0%)	6 (50.0%)	6 (50.0%)	6 (50.0%)
THC-COOH/Creatinine Ratio (ng/mg)	**Mean ± SD**	**Mean ± SD**	**Mean ± SD**	**Mean ± SD**
	-	12.00 ± 3.29	12.17 ± 4.96	11.33 ± 5.54

^a^ Limit of detection of in-house urine cup = 50 ng/mL.

**Table 3:** Cognitive performance over 6 weeks of treatment with a high-CBD product: linear mixed models (2-tailed).

	Mixed Model	Baseline *n* = 12	6 Weeks *n* = 12
	Fixed Effects	Mean ± SD	Mean ± SD
**Multi-Source Interference Task (MSIT)**			
Control Response Time (ms)	***F*(1,11) = 8.204, *p* = 0.015**	579.00 ± 69.45	542.73 ± 57.13
Control Accuracy (%)	***F*(1,11) = 6.557, *p* = 0.026**	98.79 ± 1.07	99.74 ± 0.47
Interference Response Time (ms)	***F*(1,11) = 26.694, *p* < 0.001**	873.74 ± 84.81	828.06 ± 81.99
Interference Accuracy (%)	***F*(1,11) = 15.775, *p* = 0.002**	88.37 ± 6.65	95.06 ± 3.42
**Stroop Color Word Test**			
Interference Time (s)	*F*(1,11) = 1.798, *p* = 0.207	95.33 ± 26.41	91.92 ± 21.34
Interference Accuracy (%)	*F*(1,11) = 0.186, *p* = 0.674	98.17 ± 2.44	97.92 ± 2.23
**Trail Making Test (TMT)**			
Trails A Time (s)	***F*(1,11) = 5.872, *p* = 0.034**	28.50 ± 9.24	23.50 ± 4.56
Trails B Time (s)	*F*(1,11) = 4.066, *p* = 0.069	60.92 ± 21.47	49.25 ± 22.00
**Letter-Number Sequencing (LNS)**			
LNS Total	*F*(1,11) = 0.011, *p* = 0.919	12.67 ± 2.77	12.75 ± 2.80
**Controlled Oral Word Association Task (COWAT)**			
Phonemic Fluency	*F*(1,11) = 0.006, *p* = 0.938	44.17 ± 9.71	44.33 ± 7.92
**Benton Visual Retention Task (BVRT)**			
BVRT Total	*F*(1,11) = 1.941, *p* = 0.191	7.42 ± 1.51	7.92 ± 1.38
**Rey Auditory Verbal Learning Task (RAVLT)**			
Trials 1–5: Total Correct	*F*(1,11) = 0.004, *p* = 0.953	49.92 ± 10.33	50.08 ± 7.08
Trials 1–5: Total Perseverations	*F*(1,11) = 4.538, *p* = 0.057	3.58 ± 3.23	2.00 ± 2.04
Short Delay: Correct	*F*(1,11) = 2.883, *p* = 0.118	9.25 ± 3.77	10.75 ± 2.83
Short Delay: Perseverations	*F*(1,11) = 0.186, *p* = 0.674	0.25 ± 0.45	0.17 ± 0.39
Long Delay: Correct	*F*(1,11) = 0.100, *p* = 0.758	9.67 ± 3.85	10.00 ± 2.73
Long Delay: Perseverations	*F*(1,11) = 0.314, *p* = 0.586	0.17 ± 0.39	0.08 ± 0.29

Significant contrasts are noted in **bold** (*p* ≤ 0.050)

## Data Availability

The dataset and associated documentation for the current study are available from the corresponding author under a data-sharing agreement. The data sharing agreement will provide for commitment and agreement for the following: (1) to use the data only for Institutional Review Board (IRB)-approved research purposes; (2) to not attempt to re-identify the data; (3) to not share data with any other person or entity without permission from the investigator; (4) to secure the data using appropriate secure computer technology; and (5) to destroy or return the data after analyses are completed.

## Supplementary Materials

The following supporting information can be downloaded at: https://www.mdpi.com/article/10.3390/biomedicines13081874/s1, Table S1: Clinical Changes Over 6 Weeks of Treatment with a High-CBD Product: Linear Mixed Models (2-Tailed).

## Abbreviations

The following abbreviations are used in this manuscript:

ALR	Abuse Liability Rating
BAI	Beck Anxiety Inventory
BDI	Beck Depression Inventory
BID	Twice daily
BVRT	Benton Visual Retention Test
CBD	Cannabidiol
COWAT	Controlled Oral Word Association Test
CW	Charlotte’s Web
D9-THC	Delta-9-tetryhydrocannabinol
GC-MS	Gas chromatography-mass spectrometry
HAM-A	Hamilton Anxiety Rating Scale
LC	Liquid chromatography
LMM	Linear mixed model
LNS	Letter-Number Sequencing
MC	Medical cannabis
MCT	Medium chain triglyceride
MSIT	Multi-Source Interference Test
OASIS	Overall Anxiety Severity and Impairment Scale
PANAS	Positive and Negative Affect Scale
PGIC	Patient’s Global Impression of Change
POMS	Profile of Mood States
PSQI	Pittsburgh Sleep Quality Index
RAVLT	Rey Auditory Verbal Learning Test
SAD	Social anxiety disorder
SCID	Structured Clinical Interview—DSM-5
SEQ	Side Effects Questionnaire
SF-36	Medical Outcomes Study Questionnaire Short Form 36 Item
SPST	Simulated public speaking test
SSRI	Selective serotonin reuptake inhibitor
STAI	State-Trait Anxiety Inventory
TID	Three times per day
TMD	Total Mood Disturbance
TMT	Trail Making Test
U.S.	United States
WAIS-R	Wechsler Adult Intelligence Scale-Revised
WASI-II	Wechsler Abbreviated Scale of Intelligence—II
