# Supplementary material for: Clinical and Cognitive Improvement Following Treatment with a Hemp-Derived, Full-Spectrum, High-Cannabidiol Product in Patients with Anxiety: An Open-Label Pilot Study
Source: Biomedicines. 2025 Aug 1;13(8):1874. doi: 10.3390/biomedicines13081874 (PMC12383689; doi:10.3390/biomedicines13081874)
Supplement: Supplementary file 1 [file biomedicines-13-01874-s001.zip › biomedicines-3750725-supplementary.pdf]

Table S1. Clinical Changes Over 6 Weeks of Treatment with a High-CBD Product: Linear Mixed Models (2-Tailed)

	Mixed Model	Baseline n=12	1 Weeks n=12	2 Weeks n=12	3 Weeks n=12	4 Weeks n=12	5 Weeks n=11	6 Weeks n=12
	**Fixed Effects**	**Mean±SD**	**Mean±SD**	**Mean±SD**	**Mean±SD**	**Mean±SD**	**Mean±SD**	**Mean±SD**
**Anxiety Scales**								
BAI	**F(1,6)=11.676, p<.001**	16.67±5.60	7.92±4.01***	5.50±3.29***	5.25±3.52***	6.33±55.10***	5.73±5.27***	5.00±4.02***
OASIS	**F(1,6)=9.284, p<.001**	9.92±2.35	6.75±2.49***	5.58±2.64***	4.33±1.87***	4.42±1.98***	4.00±2.24***	4.50±2.54***
HAM-A	**F(1,6)=38.400, p<.001**	18.58±5.18	8.58±3.34***	7.17±3.33***	5.00±2.56***	4.83±2.69***	4.64±3.14***	4.17±2.76***
STAI-State	**F(1,6)=2.694, p=.022**	52.08±14.86	44.17±9.81***	42.75±10.57**	42.25±11.32**	41.75±12.09*	37.82±11.29**	41.00±13.09*
STAI-Trait	**F(1,6)=3.490, p=.005**	50.67±9.86	47.00±9.18**	44.50±10.54***	42.33±10.44***	42.17±10.03***	39.18±10.98***	41.83±13.06**
**Mood & Affect Scales**								
BDI	**F(1,6)=7.184, p<.001**	14.17±7.23	7.75±6.03***	6.67±5.90***	6.67±5.94***	5.33±3.85***	4.64±4.72***	6.17±6.31***
POMS TMD	**F(1,6)=2.826, p=.017**	48.67±26.89	37.33±22.94**	32.25±30.62**	23.58±28.16***	23.42±30.32***	18.45±33.16***	20.92±32.73**
PANAS Positive	**F(1,6)=3.736, p=.003**	31.92±6.39	34.67±7.39**	33.58±8.96	35.67±7.67*	34.75±8.13	34.73±8.88	35.67±9.09
PANAS Negative	**F(1,6)=11.594, p<.001**	22.08±5.95	14.75±3.25***	14.50±4.30***	13.83±3.79***	12.83±3.13***	13.55±4.82***	12.67±2.53***
**Sleep Scales**								
PSQI	**F(1,11)=5.795, p=.035**	7.50±3.94	-	-	-	-	-	4.75±3.22
**Quality of Life Scales**								
SF36 Physical Function	F(1,11)=1.774, p=.210	95.83±7.02	-	-	-	-	-	97.92±2.57
SF36 Physical Health	F(1,11)=1.692, p=.220	83.33±26.83	-	-	-	-	-	91.67±16.28
SF36 Emotional Problems	**F(1,11)=9.428, p=.011**	36.11±38.82	-	-	-	-	-	69.44±41.34
SF36 Energy/Fatigue	**F(1,11)=40.857, p<.001**	35.00±15.95	-	-	-	-	-	56.67±18.13
SF36 Emotional Well-Being	**F(1,11)=18.692, p=.001**	54.67±12.57	-	-	-	-	-	72.67±13.30
SF36 Social Function	**F(1,11)=13.750, p=.003**	61.46±12.45	-	-	-	-	-	82.29±15.50
SF36 Pain	F(1,11)=0.534, p=.480	77.92±18.34	-	-	-	-	-	82.08±17.58
SF36 General Health	F(1,11)=4.104, p=.068	68.75±18.36	-	-	-	-	-	75.00±16.92

Bold numbers are significant at Bonferroni-corrected p≤.025, *Italicized* numbers are findings that did not survive Bonferroni correction p≤.050.

Significant least significant difference (LSD) post hoc contrasts between baseline and follow-up visits are noted as $^{*}p$≤.050; $^{**}p$≤.010; $^{***}p$≤.001
